# 

**Published:** 1908-09-15

**Authors:** 


CONTENTS

Event and Comment - - - - - - - - - 433

Metallic Posts Enlarged to Fit Badly Decayed Root Canals,
*By R. J. Cigrand, M.D., D.D.S.* 434

Orthodontia, its Significance to the General Dentist,
*By James D. Locke, D.D.S.* 449

Pyorrhea Alveolaris and its Treatment,
*By A. J. Macdonagh, D.D.S.* 456

Report of Committee on Alloys,
*By Drs. G. S. Allen, A. H. Merritt and J. Morgan Howe* 467

Indents - - - - - - - - - - - 470

Announcements - - - - - - - - - - 481

